# Viscoelastic Particle Encapsulation Using a Hyaluronic Acid Solution in a T-Junction Microfluidic Device

**DOI:** 10.3390/mi14030563

**Published:** 2023-02-27

**Authors:** Anoshanth Jeyasountharan, Francesco Del Giudice

**Affiliations:** Department of Chemical Engineering, School of Engineering and Applied Science, Faculty of Science and Engineering, Swansea University, Swansea SA1 8EN, UK

**Keywords:** droplet microfluidics, viscoelasticity, non-newtonian liquids

## Abstract

The encapsulation of particles and cells in droplets is highly relevant in biomedical engineering as well as in material science. So far, however, the majority of the studies in this area have focused on the encapsulation of particles or cells suspended in Newtonian liquids. We here studied the particle encapsulation phenomenon in a T-junction microfluidic device, using a non-Newtonian viscoelastic hyaluronic acid solution in phosphate buffer saline as suspending liquid for the particles. We first studied the non-Newtonian droplet formation mechanism, finding that the data for the normalised droplet length scaled as the Newtonian ones. We then performed viscoelastic encapsulation experiments, where we exploited the fact that particles self-assembled in equally-spaced structures before approaching the encapsulation area, to then identify some experimental conditions for which the single encapsulation efficiency was larger than the stochastic limit predicted by the Poisson statistics.

## 1. Introduction

The compartmentalisation or encapsulation of objects in picolitres droplets is a widely employed process across a variety of applications [1,2,3,4,5], including the screening of antibodies [6], enzymes [7] and proteins [8,9], as well as other cell-to-cell interaction analysis [10]. The compartmentalisation of particles in droplets is generally obtained within microfluidic devices, as they allow a precise control of the experimental parameters required to produce droplets having a uniform constant size [11,12,13]. The encapsulation process is governed by the same principles behind the formation of droplets, with a dispersed phase containing the particles that ‘meet’ a continuous non miscible phase at a junction in order to form a droplet [12,13]. The junction at which the two fluids meet can present a T-junction, a flow-focusing or a concentric geometry [11,12,13]. The actual encapsulation efficiency is instead governed by the so-called Poisson limit [14,15], which for the encapsulation of one particle per droplet is around 37%, meaning that the remaining droplets will either be empty or contain more particles in the same droplet. The reason for the existence of such limit is the fact that particles approaching the encapsulation area do not arrive at a constant frequency, at variance with droplets that are instead generated at a constant frequency governed by the volumetric flow rate values of both continuous and dispersed liquid phase [12]. The requirement of improving the encapsulation efficiency above the Poisson limit has attracted significant interest in the recent years.

Edd et al. [14] were the first to introduce a methodology to overcome the Poisson limit by using the particle ordering phenomenon [16,17], where particles were equally-spaced on one or more streamlines before approaching the encapsulation area. In general, when flowing particles are equally-spaced, the frequency of particles approaching the encapsulation area becomes constant [15]: the authors synchronised this frequency to the one of droplet formation, thus achieving a single encapsulation efficiency above the Poisson limit. Several other works [15,18,19,20,21,22,23] have followed this original idea and designed microfluidic devices to take advantage of the inertial particle ordering phenomenon [16,17,24,25,26] to overcome the Poisson statistic limit.

Very recently, the same principle originally introduced by Edd et al. [14] was employed within the framework of particle encapsulation using non-Newtonian viscoelastic liquids [27,28]. In these works, the authors took advantage of the recently discovered viscoelastic particle ordering phenomenon [29,30,31,32,33,34] to demonstrate a viscoelastic particle encapsulation up to 2-folds larger than the Poisson limit. In one case [27], the authors employed a commercial T-junction glass microfluidic devise together with an aqueous shear-thinning xanthan gum suspending liquid. In another case [28], the authors designed a microfluidic device with a flow-focusing configuration and employed aqueous solutions of hyaluronic acid, demonstrating viscoelastic encapsulation and co-encapsulation of particles up to 2-folds larger than the Poisson limit. One key advantage of using non-Newtonian rather than Newtonian liquids to equally-space particles is the fact that viscoelastic ordering is achieved on a single-line only (instead of the multiple line ordering often observed for inertial ordering [16]), thus leading to the determination of a predictive formula to achieve controlled encapsulation, as reported previously [27,28]. The encapsulation of particles in viscoelastic liquids is more difficult to achieve compared to the Newtonian case, because the elasticity of the fluids can hinder the droplet formation phenomenon or lead to a flow instability [35,36,37,38]. To this day, the two manuscripts above [27,28] are the only ones featuring viscoelastic encapsulation of particles, with several questions remaining unanswered. For instance, the suspending liquids employed so far were not appropriate to work with cells, as cells require phosphate buffer saline (PBS) in order to survive. The addition of PBS to the suspending liquids reported in the previous works, however, could result in changes of their rheological properties, notably, a reduction in the magnitude of the shear-thinning [39,40], which in turn would lead to the suppression of particle ordering, in favour of particle string formation (i.e., particles attached to each other) [29,31,32]. Furthermore, the devices introduced so far were still affected by the problem of particle aggregates (e.g., doublets or triplets) that were breaking the continuity of the particle train [30,32,41].

In this work, we employed a hyaluronic acid solution in PBS to study the viscoelastic encapsulation of particles in a T-junction microfluidic device. We chose hyaluronic acid as viscoelastic liquid because of its high biocompatibility when compared to other polymer solutions [42], and because it has successfully been used recently to demonstrate controlled particle encapsulation in flow-focusing geometries [28]. Moreover, hyaluronic acid is a more stable polymer compared to the xanthan gum previously employed, which tends to quickly degrade in solution over time. At variance with standard and commercially available T-junction devices, our microfluidic device featured the addition of several trapezoidal elements required to break the particle aggregates, as recently demonstrated in another work [43]. We first studied the viscoelastic droplet formation mechanism and then the viscoelastic encapsulation. We identified some optimal experimental conditions for which the single particle encapsulation efficiency was larger than the Poisson limit.

## 2. Materials and Methods

### 2.1. Microfluidic Device Design and Fabrication

**Figure 1:**
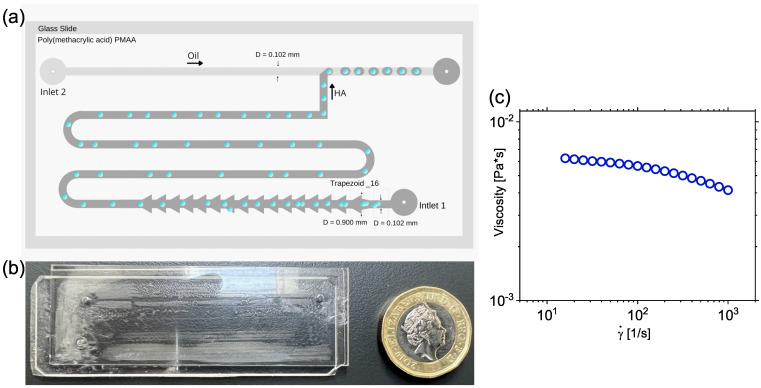
(**a**) Schematic representation of the T-junction device employed in this work. The dispersed viscoelastic phase entered the device via Inlet 1. The trapezoidal structures were added in order to break down potential particle aggregates, in agreement with previous works [43]. After the trapezoidal structure, particles first aligned on the channel centreline and then self-ordered before approaching the encapsulation area. The continuous phase entered the device via Inlet 2, and the droplets containing flowing particles were formed at the T-junction. (**b**) Image of the Microfluidic device employed in this study next to a 1 pound coin. The device was made of Polymethylemethacrylate bonded on a glass slide using a double-sided tape (see main text for more details) (**c**) Shear viscosity η as a function of the shear rate $\dot{\gamma }$ for the hyaluronic acid solution at 0.1 wt% in phosphate buffer saline. The solutions displays shear-thinning properties above a value of the shear rate equal to $\dot{\gamma }≃30\;s^{-1}$.

Experiments were performed in a square shaped microfluidic device designed to facilitate the encapsulation of particles with efficiency above the stochastic Poisson value (Figure 1a,b). Particles suspended in the polymer solution entered the microfluidic device and then moved through a series of trapezoidal elements, similar to the ones originally reported by Liu et al. [30]. When entering a trapezoidal element, particles first slowed-down because of a change in cross-section, to then accelerate when leaving: the sudden acceleration facilitated separation among particles. Jeyasountharan et al. [43] employed the original concept introduced by Liu et al. [30] to quantify the break up efficiency of aggregates entering the microfluidic device. Building on the previous results, here the particles entered the channel via Inlet 1 where they went through 16 trapezoidal elements where potential aggregates could be broken down to individual particles. We did not quantify here the particle breakdown efficiency, as this was already reported in our previous work [43], where more than 90% of isolated particles were observed at the end of the trapezoidal structures. Afterwords, particles travelled along the serpentine channel to facilitate the formation of equally-spaced particle trains, as reported previously [29,41]. The continuous phase entered the microfluidic device via Inlet 2 (Figure 1a), and it met the dispersed phase at the T-junction, leading to the formation of a droplet containing encapsulated particles (Figure 1a). We chose a T-junction geometry because it is linked to several applications related to biosynthesis [44], drug delivery [45] and microgel fabrication [46]. Additionally, we wanted to test the hyaluronic acid with the addition of PBS as a suspending fluid for controlled encapsulation applications, being the previous study on T-junction focused on the use of xanthan gum [27], while the other one on flow-focusing geometries featured hyaluronic acid without PBS [28].

A micromilling machine (Minitech CNC Mini-Mill) was used to fabricate the microfluidic device on a rigid polymethylmethacrylate (PMMA) substrate with a thickness of 1.2 mm, using the same approach employed in previous works [28]. Firstly, a 2-mm-wide tip was used to mill the substrate down to 300 μm to obtain a uniform surface. A 100 μm metal tip was used to mill the channel onto the PMAA substrate. Then, a 0.5-mm-wide tip was used to mill the inlet and outlet holes, which had a diameter of 1.6 mm. The channel depth was kept constant to 100 μm and the width of the square shaped microfluidic device was equal to 100 μm. Following device fabrication, the PMMA substrate was placed in an ultrasonic bath for 30 min to remove excess material. Pressurized air was used to fully dry the PMMA substrate. A double-sided tape (Adhesives Research, Limerick Ireland) was employed to bond the PMMA substrate onto a glass slide. Home-made adapters were attached to the two inlets of the microfluidic device using a double-sided tape (Adhesives Research, Limerick Ireland). PMMA has been identified in the past as a source of potentially unstable droplet generation [47], thus requiring surface modification. In our case, however, as also in our previous study featuring controlled encapsulation in a flow-focusing geometry [28], we observed stable droplet generation. The lack of unstable droplet formation in PMMA devices echoed some previous works available in the literature [48,49,50].

### 2.2. Sample Preparation and Characterization

A solution of hyaluronic acid salt from *Streptococcus equi* (HA, Sigma Aldrich, UK) having molecular weight in the range 1.5–1.8 MDa at a mass concentration of 0.1 wt% in phosphate buffer saline (PBS, Sigma Aldrich UK) was used as the dispersed phase in all the experiments on viscoelastic flows. The HA concentration was chosen such that the fluid still presented shear-thinning properties, but the zero-shear viscosity was not too large to affect particle mixing. In terms of molecular conformation, the polymer concentration employed here was far from displaying any polymer entanglement, as it has been previously showed that HA at similar molecular weight dispersed in phosphate buffer saline falls either in the dilute or in the semi-dilute unentangled regime [39,51,52]. The polymer powder was added directly to the PBS, and the resulting solution was stirred with a magnetic stirrer (Fisherbrand) for 12 h to allow full dissolution of the polymer. A stress-controlled rheometer (TA AR2000ex) with a truncated acrylic cone (60 mm diameter, 1° angle) was used for the rheological measurements at a constant temperature of T=22 °C. The fluid presented a near constant-viscosity region for shear rate values $\dot{\gamma }\leq 30\;s^{-1}$, while exhibiting shear-thinning properties at larger shear-rate values (Figure 1c). The presence of shear-thinning is very important to obtain particle trains, as liquids displaying a near constant-viscosity are more likely to lead to string of attached particles rather than trains of equally-spaced objects [29,53]. To quantify the fluid elasticity, we attempted to measure the longest relaxation time λ of the solution via conventional small angle oscillatory shear (SAOS) measurements; however, the rheometer was not sufficiently sensitive to detect such small values of λ. For this reason, we determined λ by using the μ-rheometer microfluidic rheometer recently introduced [54,55], obtaining a value of λ=7.27±1.73 ms.

Mineral oil (Sigma Aldrich) was used as the continuous phase in all the experiments. Span 80 (Sigma Aldrich, UK), a non-ionic surfactant, was added at a concentration of 1 wt% to the oil to stabilize the interface between HA and oil, in agreement with previous works [27,28]. The viscosity of the mineral oil taken from the same batch employed here was previously measured in our lab and found to be 29 mPa·s [27,28]. The interfacial tension values between 0.1 wt% HA in PBS and mineral oil was here measured using a force tensiometer (Sigma 702, Dyne Testing, Lichfield, UK) equipped with a du Nouy ring. In this methodology, the du Nouy ring was first submerged in the HA solution (heavier phase), while mineral oil (lighter phase) was poured on top. The ring was brought into contact with the interface using the microbalance embedded in the instrument and the interfacial tension was obtained by measuring the force required to separate the ring from the interface. The measured value of interfacial tension was γ=3.35±0.13 mN/m, in line with the values previously obtained for aqueous HA solutions in contact with the mineral oil [28], thus suggesting that the presence of the PBS had no effect on the interfacial tension value.

For the encapsulation experiments, polystyrene particles (Polysciences) with diameter of 20±2μm were added to the 0.1 wt% HA polymer solution in PBS at a volumetric concentration ϕ=0.2 vol%: this is in agreement with previous studies [28], where the authors employed 20μm particles flowing in a 100μm microchannel to trigger particle ordering, thus opening the way towards controlled particle encapsulation. The suspension was mixed in a vortex mixer (Fisherbrand ZX3) to fully disperse the polystyrene particles in the HA polymer solution. Aggregates were removed by placing the solution in a ultrasonic bath for 2 min.

### 2.3. Experimental Apparatus and Particle Tracking

The microfluidic device was connected to a glass syringes (Hamilton) having a 1/4-28 male thread via a 10-cm long fluorinated ethylene propylene tube (Dolomite Microfluidics) with an external diameter of 1.6 mm and an internal diameter of 0.25 mm. The tubing was connected to a syringe pump (KD Scientific). The flow rate values of the continuous phase was kept constant at either Q=10, 8, 6, 4, 2 or 1 μL/min. For these values of the volumetric flow rate, the Capillary number $Ca=\mu_{c}U_{c}/\gamma$, where $U_{c}=Q_{c}/\left(WH\right)$ is the average velocity of continuous phase fluid, $\mu_{c}$ is the viscosity of the continuous phase and γ is the interfacial tension, was in the region Ca∈ [0.01–0.1]. The flow rate of dispersed phase for droplet generation and encapsulation experiments was varied in the range Q= 10, 8, 6, 4, 2 or 1 μL/min for each constant value of oil flow rate.

An inverted microscope (Zeiss Axio A1) connected to a fast camera (Photron Mini UX50) was used to acquire all the videos of droplet formation and particle encapsulation. Videos were captured at a frame rate of 250 fps. The acquired videos were analysed using a home-made code written in Matlab to determine size and frequency of droplet generation. For the particle encapsulation experiments, the number of particle per each droplet was counted manually.

## 3. Results and Discussion

### 3.1. Droplet Formation

**Figure 2:**
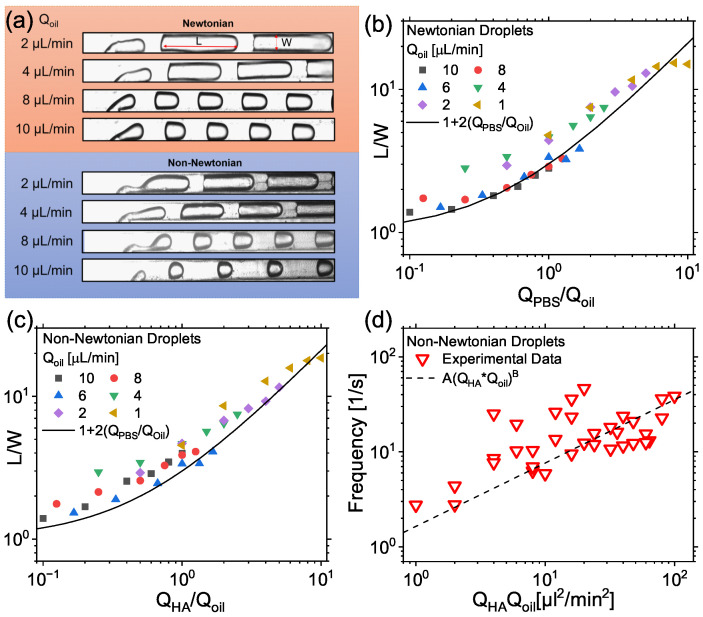
Droplet generation formation and scaling in a T-junction microfluidic device. (**a**) Experimental snapshots of droplet generation at various continuous phase (oil) flow rates $Q_{oil}$. For the Newtonian case, phosphate buffer saline (PBS) is employed as the dispersed phase. For the non-Newtonian case, hyaluronic acid (HA) at a mass concentration of 0.1 wt% is used as dispersed phase. Satellite droplet formation is only observed in the non-Newtonian case. The volumetric flow rate of the dispersed phase is 8μL/min in both cases. (**b**,**c**) Normalised droplet size L/W, where *L* is the droplet length and W=100μm is the channel width (see experimental snapshot in (**a**)), as a function of the ratio $Q_{PBS}$/$Q_{oil}$ for the Newtonian droplets (**b**) and $Q_{HA}$/$Q_{oil}$ for the non-Newtonian droplets (**c**). $Q_{PBS}$ and $Q_{HA}$ are the flow rates of PBS and HA, respectively, while $Q_{oil}$ is the flow rate of the mineral oil. The solid line in (**b**,**c**) is $L/W=1+2\left(Q_{PBS}/Q_{oil}\right)$, meaning that the non-Newtonian data in (**c**) collapse on the master curve for Newtonian droplets. (**d**) Frequency of non-Newtonian droplet generation as a function of the product $Q_{HA}Q_{oil}$. Data points collapse on the master curve $f_{drop}=A{\left(Q_{HA}\times Q_{oil}\right)}^{B}$ with A=1.64±0.18 and B=2/3. The parameter *A* was obtained by fitting the entire data set with flow rate values in the units of μL/min, while *B* was fixed to B=2/3 according to the previously introduced by Shahrivar and Del Giudice [27] for xanthan gum solutions.

In this manuscript, we studied the encapsulation of particles in viscoelastic liquids in a T-junction microfluidic device, with the ultimate aim of identifying experimental conditions for which the encapsulation efficiency was larger than the Poisson limit. In analogy with previously published works on the topic [27,28], we first studied the viscoelastic droplet formation phenomenon at various imposed flow rate ratios $Q_{d}/Q_{c}$, where $Q_{d}$ is the flow rate of the dispersed phase (i.e., either PBS or HA) and $Q_{c}$ is the flow rate of the continuous mineral oil phase (Figure 2). Droplet formation was very stable during our experiments, and we did not observe any unstable behaviour even though the microchannel surface remained untreated, in agreement with the experimental setup employed previously elsewhere [28].

Under the qualitative point of view (Figure 2a), for the same set of imposed volumetric flow rate values, the droplet size did not differ significantly between Newtonian and non-Newtonian droplets, in agreement with previous finding on viscoelastic droplet microfluidics [27,28,35,36,37,56,57,58,59]. However, we observed a clear difference in the dynamics of droplet formation between Newtonian and non-Newtonian droplets, with the presence of satellite droplet formation only observed for the non-Newtonian case (Video S1), in agreement with previous findings featuring formation of viscoelastic droplets made of aqueous xanthan gum solutions in a commercial T-junction device [27]. It has been previously demonstrated [27,28,59,60] that Newtonian and non-Newtonian droplets in both T-junction and flow-focusing geometries could display a universal scaling, when plotting the droplet length *L* normalised by the channel width *W* as a function of the ratio between the volumetric flow rate of the continuous phase and that of the dispersed phase. In analogy with this approach, we also quantified the droplet length *L* normalised by the channel width W=100μm, as a function of the ratio between $Q_{PBS}$ and $Q_{oil}$ (Figure 2b,c). For Newtonian droplets (Figure 2b), we observed that all the data scaled on a mastercurve given by the expression $L/W=1+2\left(Q_{PBS}/Q_{oil}\right)$, in agreement with previous findings [60]. Similarly, we observed that the normalised droplet length for non-Newtonian droplets scaled identically to the Newtonian case (Figure 2c), again reinforcing the qualitative observations in Figure 2a, and previous findings on viscoelastic droplet microfluidics [27,28,35,36,37,56,57,58,59]. In addition to the normalised droplet size, we also quantified the frequency of droplet formation (Figure 2d). This is a very important step in the pursue of controlled encapsulation [14,15,27,28], because the Poisson stochastic limit can be overcome only when the frequency of droplet formation $f_{d}$ is equal to the constant frequency of equally-spaced particles $f_{p}$ approaching the encapsulation area. We observed that our experimental data for the droplets made of HA in PBS scaled according to $f_{d}=A{\left(Q_{HA}Q_{oil}\right)}^{B}$ with A=1.64±0.18 and B=2/3. The parameter *A* was obtained by fitting the entire data set with flow rate values in the units of μL/min, while *B* was fixed to B=2/3 according to the scaling previously introduced by Shahrivar and Del Giudice [27] for xanthan gum solutions. The only difference was the value of the parameter *A*, which simply corresponded to a vertical shift. Such discrepancy can be due to a variety of factors, including the different rheological properties between HA and xanthan gum, or the fact that the microfluidic device employed by Shahrivar and Del Giudice [27] was made of glass with a hydrophobic coating, while here we employed PMMA bonded to glass via an adhesive tape. A qualitative argument to explain the scaling of the droplet frequency with the product of the two volumetric flow rates was introduced previously [27], where the authors speculated that an increase in the volumetric flow rate of the continuous phase led to strong shear and drag forces acting on the viscoelastic filament, thus causing a faster droplet pinch-off. Our results seem to support the previous argument.

Taken together, the data on droplet formation suggested that, while the dynamics of droplet formation was different between the Newtonian and non-Newtonian case, the droplet size remained substantially unchanged. Furthermore, the data related to the frequency of droplet formation scaled with the same exponent as the data presented previously [27].

### 3.2. Viscoelastic Encapsulation of Particles

**Figure 3:**
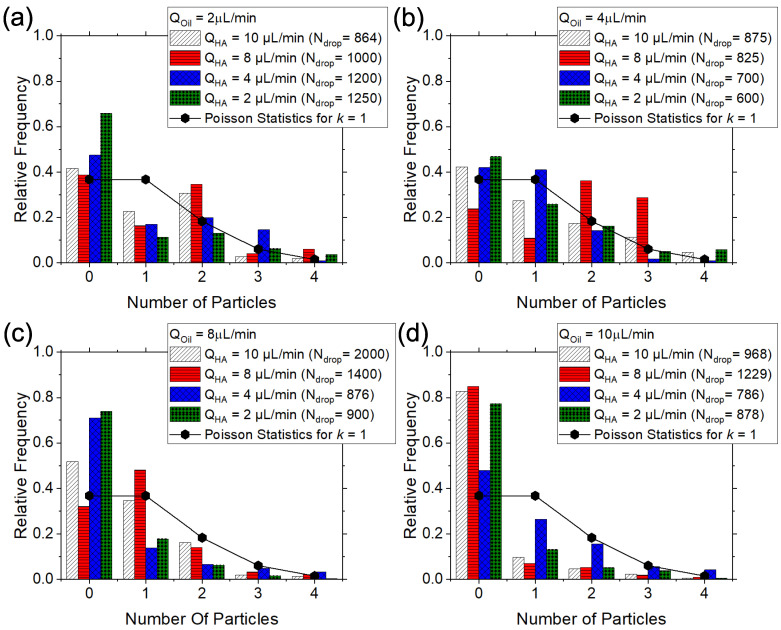
Viscoelastic particle encapsulation for a hyaluronic acid (HA) dispersed phase and a mineral oil continuous phase. (**a**–**d**) Histograms of relative frequency as a function of particles per droplet for a fixed oil flow rate $Q_{oil}$. For each fixed $Q_{oil}$ value, the flow rate of the HA $Q_{HA}$ was in the range 2 to 10 μL/min. The Poisson statistics value obtained for k=n=1 is represented by the solid symbols. A single particle encapsulation efficiency above the Poisson stochastic value is obtained for $Q_{oil}$ = $Q_{HA}=4\;\mu$L/min (**b**) and $Q_{oil}$ = $Q_{HA}=8\;\mu$L/min (**c**).

After characterising the droplet generation phenomenon, we studied the viscoelastic encapsulation of particles in the T-junction device (Figure 3).

For a purely stochastic encapsulation, the probability P(k,n) of encapsulating a given number of particles *n* in a droplet is expressed by $P\left(k,n\right)=k^{n}exp\left(-k\right)/\left(n!\right)$, where *k* is the average number of particles per droplet [15,61]. One of the most desired conditions is the encapsulation of 1 particle in 1 droplet, meaning a value of number of encapsulated particles n=1 and an average number of particles per droplets k=1 (solid symbols connected by lines in Figure 3). This result is difficult to achieve without significantly diluting the solution containing the particles at a cost of having several empty droplets, resulting in an overall encapsulation efficiency quantified by the Poisson statistics [15]. To achieve an encapsulation efficiency larger than the Poisson statistics, it has recently been showed that polymer solutions can be used as a suspending liquid for the particles [27,28]. Indeed, at a sufficiently large local particle concentration required for the particles to experience hydrodynamic interactions among nearby particles, the viscoelasticity of the fluid drove the formation of equally spaced particles (also called particle trains) on the centreline of a microfluidic channel. Once that the distance between particles became constant, the frequency of particles approaching the encapsulation area was also constant and it could be synchronised to the frequency of droplet formation in order to achieve the desired result of 1 particle in 1 droplet without reducing the particle concentration in the suspension. Such approach has been previously limited by the formation of particle aggregates that disturbed the continuity of the particle train. To address this problem, Jeyasountharan et al. [43] have previously demonstrated that a sequence of trapezoidal elements located after the channel inlet could significantly reduce the occurrence of particle aggregates; the role of the trapezoidal structures was only found to break the agglomerates without leading to particle ordering, as the length required for the particle to self-assemble in a continuous particle train are longer. In this work, we adopted the same approach, and we observed that the majority of particles approaching the encapsulation area were isolated (see for instance Video S2), in agreement with previous works [43]. We compared our viscoelastic encapsulation data against the stochastic Poisson value to see whether we could overcome the Poisson stochastic limits by taking advantage of the particle ordering phenomenon, similarly to previous studies on viscoelastic [27,28] or inertial [14,15] encapsulation. Particles suspended in the shear-thinning HA solution in PBS were expected to equally-space ahead of the encapsulation area thanks to the viscoelasticity-mediated hydrodynamic interactions between consecutive particles [27,28,29], meaning that they would approach the T-junction at a constant frequency. For a volumetric flow rate of the continuous phase $Q_{Oil}=2\;\mu$L/min, we observed that the viscoelastic single encapsulation efficiency was always lower than the Poisson limit (Figure 3a). By increasing the volumetric flow rate of the dispersed phase to $Q_{Oil}=4\;\mu$L/min, we observed a slight improvement of encapsulation efficiency over the Poisson limit for $Q_{HA}=4\;\mu$L/min (Figure 3b). The situation improved greatly when $Q_{oil}=Q_{HA}=8\;\mu$L/min, as we observed an encapsulation efficiency value of around 50%, significantly larger than the Poisson value at around 36% (Figure 3c, Video S2). When increasing the flow rate of oil to $Q_{Oil}=10\;\mu$L/min, we could not identify any value for the HA flow rate for which the encapsulation efficiency was above the Poisson limit. With reference to the same set of data for $Q_{Oil}=8\;\mu$L/min, there was only one value of the hyaluronic acid flow rate ($Q_{HA}=8\;\mu$L/min) for which the encapsulation efficiency was larger than the Poisson stochastic limit. This is not surprising, as the whole principle of overcoming the Poisson limit is based on the fact that the frequency of droplet formation $f_{d}$ needs to be synchronised to the one of particles approaching the encapsulation area $f_{p}$. The frequency of droplet formation was always constant for the whole duration of the experiments and its value was controlled by the two volumetric flow rates, that of the dispersed phase (HA) and that of the continuous phase (mineral oil). In an ideal particle train, i.e., where all the particles are equally-spaced at the same constant distance from each other, the frequency $f_{p}$ would be constant; however, since we observed fluctuations in the local particle concentrations in agreement with previous works [16,27,41], the value of the frequency $f_{p}$ was not entirely constant, thus leading to a distribution of particle frequencies. This means that the particle frequency would only match the droplet frequency when the distance between consecutive particles would be such that the resulting frequency was equal to $f_{d}$. In the total absence of particle ordering, where there is no dominant inter-particle distance, particles will either be encapsulated according to the Poisson stochastic limit or with even lower encapsulation values (caused by the encapsulation of multiple particles in the same droplet). Thanks to the particle ordering, a characteristic frequency of particle approaching the encapsulation area (set by the local particle concentration and by geometrical constraints [29,43]) was present and it could then be synchronised to the constant droplet frequency thus leading to the observation of single-particle encapsulation above the stochastic value, in agreement with previous works [14,27,28].

In terms of encapsulation efficiency, our results are not far from those derived previously [27,28]. With reference to the work by Shahrivar and Del Giudice on viscoelastic encapsulation in a T-junction using aqueous xanthan gum solutions as the dispersed phase [27], the authors demonstrated an encapsulation efficiency in the range of 50% to 60%, not far from our results. The slightly lower encapsulation efficiency value achieved here in comparison to the work by Shahrivar and Del Giudice [27] can be due to the fact that the xanthan gum employed by the authors presented more pronounced shear-thinning properties than the fluid employed here. It has been shown via numerical simulations [31,53] and experiments [29] that the rheology of the suspending liquid is essential to drive particle train formation on the centreline of a microfluidic channel. The reduction in the shear-thinning magnitude of the HA employed here compared to previous works [28,29] was due to the addition of PBS salt: being HA a polyelectrolyte, the addition of salt alters the rheological properties of the resulting solution, especially causing a reduction in the shear-thinning properties [39,62]. Even though the PBS resulted in a reduction of the shear-thinning displayed by the solution, we could still observe particle ordering and could still achieve an encapsulation efficiency above the Poisson limit. PBS is essential when working with cells, meaning that the solutions employed here could be used for future studies on cell ordering and encapsulation. It is also worth mentioning that we still experienced fluctuations in the local concentration of particles approaching the encapsulation area, which is a common unsolved problem encountered when dealing with large particle concentrations [16,41]. Since the particle concentration was fluctuating, two consecutive particles could experience different hydrodynamic interactions depending on the distance between them, thus altering the stability and the continuity of the train, and therefore having a negative impact on the encapsulation efficiency. While no definitive solution has currently been introduced to address this problem (with the exception of the design proposed by Liu et al. [30]), we still identified areas for which the encapsulation efficiency was larger than the Poisson stochastic limit, meaning that particle ordering was beneficial to promote encapsulation with efficiency larger than the Poisson stochastic limit. This is also in agreement with a recent work featuring the controlled encapsulation of particles in flow-focusing microfluidic geometries [28].

Similarly to previous studies [27,28], we derived an expression that can be used to predict the best conditions for particle encapsulation above the Poisson limit. The frequency of particles approaching the encapsulation area can be written as $f_{p}=u\phi_{l}/d$ [27,28,29,41], where *u* is the particle velocity, *d* is the particle diameter, and $\phi_{l}$ is the local particle concentration defined as $\phi_{l}=Nd/L$ [41], where *N* is the number of particles in a given channel length (this is generally fixed by the experimental observation window) and *L* is the length of the experimental observation window. It has been showed that the local particle concentration is a better parameter to quantify the particle ordering phenomenon compared to the bulk concentration [16,41]. To overcome the Poisson stochastic limit, the frequency of droplet formation $f_{d}=A{\left(Q_{Oil}Q_{HA}\right)}^{B}$ should be equal to that of particle approaching the encapsulation area $f_{p}=u\phi_{l}/d$, thus resulting in:

(1) $$Q_{Oil}=\frac{1}{Q_{HA}}{\left(\frac{u\phi_{l}}{Ad}\right)}^{\frac{1}{B}},$$

where A=1.64±0.18 and B=2/3, with the volumetric flow rate expressed in μL/min, *d* in metres and *u* in m/s. Equation (1) is identical to the one previously introduced by Shahrivar and Del Giudice [27] for the viscoelastic controlled particle encapsulation using xanthan gum aqueous solutions; the only difference was the value of the parameter *A*, that here was around 5 times smaller compared to the one for encapsulation using xanthan gum [27]. The fact that the exponent is identical may suggest a general scaling trend for the particle encapsulation in T-junction geometries, but more work is required to demonstrate such universality.

In summary, we studied the viscoelastic encapsulation phenomenon identifying only two conditions for which the encapsulation efficiency was above the Poisson limit, namely, $Q_{Oil}=Q_{HA}=4\;\mu$L/min (with efficiency ≃40%) and $Q_{Oil}=Q_{HA}=8\;\mu$L/min (with efficiency ≃50%). We also observed that the mathematical expression to evaluate the best conditions for controlled encapsulation was identical to the one proposed earlier [27], with the only difference of a vertical shifting factor for the data.

## 4. Conclusions

In this work, we studied the viscoelastic encapsulation of particles in a T-junction microfluidic device, using hyaluronic acid 0.1 wt% in PBS as suspending liquid. We observed that the data for viscoelastic droplet formation scaled with the same scaling as the Newtonian ones, thus suggesting that the droplet size is not affected by the fluid rheology significantly, in agreement with previous works [27,28,35,36,37,56,57,58,59]. We also observed that the frequency of droplet generation scaled as $f_{d}=A{\left(Q_{HA}Q_{oil}\right)}^{B}$ with A=1.64±0.18 and B=2/3. The parameter *A* was obtained by fitting the entire data set with flow rate values in the units of μL/min, while *B* was fixed to B=2/3 according to the previously introduced by Shahrivar and Del Giudice [27] for xanthan gum solutions. The only difference was the value of the parameter *A*, which simply corresponded to a vertical shift. We also studied the viscoelastic encapsulation of particles, identifying only two conditions for which the encapsulation efficiency was above the Poisson limit, namely, $Q_{Oil}=Q_{HA}=4\;\mu$L/min (with efficiency ≃40%) and $Q_{Oil}=Q_{HA}=8\;\mu$L/min (with efficiency ≃50%). We also observed that the mathematical expression to evaluate the best conditions for controlled encapsulation was identical to the one proposed earlier [27], with the only difference of a vertical shifting factor for the data.

Future works are still required in this field. The effect of polymer entanglement in the suspending liquid on the particle encapsulation process remains not fully understood. Polymer entanglements are expected to have a significant weight in promoting faster particle self-ordering, while also potentially undermining the formation of droplets when increasing the volumetric flow rate [27]. Understanding the dynamics of droplet generation and particle/cell encapsulation has huge potential to improve several biomedical applications. For example, controlled sequences of cells in droplets with varied volumes is desirable in multi-volume droplet digital polymerase-chain-reaction (PCR) for accurate detection of genetic information in cells. In tissue engineering, droplet-based microfluidic systems are used to produce building blocks of artificial tissues and organs, such as shape-controlled micro particles and microfibers [19]. We also anticipate that new machine learning algorithms [63,64,65] have the potential to optimise the experimental parameters to improve single particle encapsulation efficiencies above the Poisson stochastic limit.

## Data Availability

All the data underpinning this research are available from the corresponding author upon reasonable request.

## Supplementary Materials

The following supporting information can be downloaded at: https://www.mdpi.com/article/10.3390/mi14030563/s1, Video S1: Formation of viscoelastic droplets for $Q_{oil}=8\;\mu$L/min and $Q_{HA}=8\;\mu$L/min; Video S2: Controlled encapsulation of particles in viscoelastic liquids for $Q_{oil}=8\;\mu$L/min and $Q_{HA}=8\;\mu$L/min.
